# Social inequalities in young children’s sports participation and outdoor play

**DOI:** 10.1186/s12966-014-0155-3

**Published:** 2014-12-16

**Authors:** Anne I Wijtzes, Wilma Jansen, Selma H Bouthoorn, Niek Pot, Albert Hofman, Vincent W V Jaddoe, Hein Raat

**Affiliations:** The Generation R Study Group, Erasmus Medical Center, Rotterdam, The Netherlands; Department of Public Health, Erasmus MC, University Medical Center, P.O.Box 2040, Rotterdam, CA 3000 The Netherlands; Department of Social Development, Rotterdam, The Netherlands; MOVE Research Institute Amsterdam, Faculty of Human Movement Sciences, VU University, Amsterdam, The Netherlands; School of Human Movement & Sports, Windesheim University of Applied Sciences, Zwolle, the Netherlands; Department of Epidemiology, Erasmus Medical Center, Rotterdam, The Netherlands; Department of Pediatrics, Erasmus Medical Center, Rotterdam, The Netherlands

**Keywords:** Socioeconomic, Ethnic, Sports, Outdoor play, Inequalities, Physical activity

## Abstract

**Background:**

Research on social inequalities in sports participation and unstructured physical activity among young children is scarce. This study aimed to assess the associations of family socioeconomic position (SEP) and ethnic background with children’s sports participation and outdoor play.

**Methods:**

We analyzed data from 4726 ethnically diverse 6-year-old children participating in the Generation R Study. Variables were assessed by parent-reported questionnaires when the child was 6 years old. Low level of outdoor play was defined as outdoor play <1 hour per day. Series of multiple logistic regression analyses were performed to assess associations of family SEP and ethnic background with children’s sports participation and outdoor play.

**Results:**

Socioeconomic inequalities in children’s sports participation were found when using maternal educational level (p < 0.05), paternal educational level (p < 0.05), maternal employment status (p < 0.05), and household income (p < 0.05) as family SEP indicator (less sports participation among low SEP children). Socioeconomic inequalities in children’s outdoor play were found when using household income only (p < 0.05) (more often outdoor play <1 hour per day among children from low income household). All ethnic minority children were significantly more likely to not to participate in sports and play outdoor <1 hour per day compared with native Dutch children. Adjustment for family SEP attenuated associations considerably, especially with respect to sports participation.

**Conclusion:**

Low SEP children and ethnic minority children are more likely not to participate in sports and more likely to display low levels of outdoor play compared with high SEP children and native Dutch children, respectively. In order to design effective interventions, further research, including qualitative studies, is needed to explore more in detail the pathways relating family SEP and ethnic background to children’s sports participation and outdoor play.

**Electronic supplementary material:**

The online version of this article (doi:10.1186/s12966-014-0155-3) contains supplementary material, which is available to authorized users.

## Background

Regular engagement in physical activity in childhood is associated with multiple physical and psychosocial health benefits, including improved academic performance, improved cardiorespiratory fitness, skeletal health, muscle strength, and motor skills, and a decreased risk of childhood overweight and obesity [1-5]. In addition to making an important contribution to overall physical activity, specific physical activity behaviors such as sports participation (team sports in particular) and unstructured play (outdoor play in particular) are assumed to bring about additional health benefits including increased social integration, teamwork and social skills, emotional control, confidence, discipline, empathy, and emotional well-being [6-8].

Studies on sports participation consistently show that children from families with a low socioeconomic position (SEP) [9-16] and ethnic minority children [9,12,17] participate less often in organized sports compared with high SEP children and ethnic majority children. Research on the associations of family SEP [13,18-20] and ethnic background [17,19] with children’s outdoor play is more scarce and conflicting, possible due to the use of different indicators of SEP [13,18-20]. Furthermore, previous research on the associations of ethnic background with children’s sports participation and outdoor play have either been conducted in the US [12,19], which hampers generalization to ethnic minority groups in Europe, or in Europe comparing heterogeneous groups of ethnic minority children (native versus non-native children [9,17]), which hampers effect evaluation for specific ethnic minority groups. As migration histories and cultural backgrounds differ substantially between children from different ethnic minority groups, these children may display very different physical activity behaviors.

In the present study, we aimed to assess the associations of family SEP, as indicated by parental educational level, parental employment status, and household income, and ethnic background with sports participation and outdoor play among 6-year-old ethnically diverse children. Data were used from the Generation R Study, a multi-ethnic prospective birth cohort in Rotterdam, the Netherlands.

## Methods

### Study design

This cross-sectional study was embedded in the Generation R Study, a population-based prospective cohort study from fetal life onwards. The Generation R Study was designed to identify early environmental and genetic determinants of growth, development and health, and has been described previously in detail [21]. The study was conducted in accordance with the guidelines proposed in the World Medical Association Declaration of Helsinki and has been approved by the Medical Ethical Committee at Erasmus MC, University Medical Center Rotterdam. Written informed consent was obtained from all participants [21].

### Study population

Invitations to participate in the study were sent out to all pregnant women who had an expected delivery date between April 2002 and January 2006 and who lived in the study area (Rotterdam, the Netherlands) at time of delivery. In total, 8305 children from the original 9749 known live born children of the Generation R cohort participate in the school aged period from 5 years onwards [21]. For the purpose of this study, we selected children born to mothers with a native Dutch, Surinamese-Creole, Surinamese-Hindustani, Dutch Antillean, Cape Verdean, Turkish, or Moroccan ethnic background (n = 6447). These ethnic groups were chosen because they represent the largest ethnic groups in the Generation R Study, as well as in the city of Rotterdam [21]. We excluded children with missing data on both sports participation and outdoor play (n = 1322). To avoid clustering of data, we furthermore excluded second (n = 392) and third children (n = 7) of the same mother, leaving a study population of 4726 participants. Of those, 4685 participants had information on sports participation and 3903 participants had information on outdoor play.

### Family socioeconomic position and ethnic background

Family SEP and ethnic background were assessed by parent-reported questionnaires when the child was 6 years old. Indicators of family SEP included maternal and paternal educational level (highest level attained), maternal and paternal employment status (no paid job, paid job), and net household income (<€2000/month (i.e. below modal income [22]), €2000-€3200/month, >€3200/month). The Dutch Standard Classification of Education was used to categorize four levels of education: low (no education, primary school, lower vocational training, intermediate general school, or three years or less general secondary school), mid-low (more than three years general secondary school, intermediate vocational training, or first year of higher vocational training), mid-high (higher vocational training) and high (university or PhD degree) [23]. Children’s ethnic background was based on the ethnic background of their mothers to take into account the cultural background of the mothers (most often primary caregivers). In accordance with Statistics Netherlands, a mother was considered nonnative Dutch if one of her parents was born abroad. If both parents were born abroad, country of birth of the mother’s mother decided on maternal ethnic background [24].

### Sports participation and outdoor play

Children’s sports participation (yes, no) and outdoor play were assessed by parent-reported questionnaire when the child was 6 years old (Additional file 1: Table S1). School-based organized activities such as physical educational lessons and swimming lessons were assessed separately and thus not included in the question on sports participation. For outdoor play, frequency (i.e. number of days) and duration (never, less than 30 minutes, 30–60 minutes, 1–2 hours, 2–3 hours, 3–4 hours) were asked for weekdays and weekend days separately. The middle number of hours of each category (e.g. 2.5 hours for 2–3 hours) was used to estimate the duration of a session. These variables were combined to estimate daily outdoor play by using the following formula: daily use = {[(days per week) * (hours on a weekday)] + [(days per weekend) * (hours on a weekend day)]}/ 7. Due to a skewed distribution, this variable was then dichotomized into <1 hour per day (i.e. low level of outdoor play) versus ≥1 hour per day.

### Potential confounders

**Figure 1:**
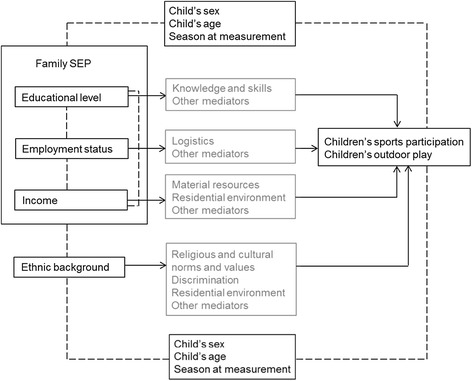
**Hypothesized interrelationships between variables included in the study.** Arrows represent hypothesized causal associations. Dotted lines represent hypothesized (non-causal) associations.

Child’s sex, child’s age, and season at measurement (summer, fall, winter, spring) were considered potential confounders in the associations of family SEP and ethnic background with children’s outdoor play and sports participation. When assessing the association between family SEP and children’s physical activity behaviors, ethnic background was considered a potential confounder, and vice versa. The hypothesized interrelationships between all variables are presented in Figure 1.

### Statistical analyses

Descriptive statistics were used to characterize the study population. Sports participation and outdoor play according to family SEP and ethnic background were evaluated by Chi-square tests. The associations of family SEP with children’s sports participation and outdoor play were assessed using series of multiple logistic regression models. The first set of models included each indicator of family SEP separately (i.e. crude models). The second set of models were adjusted for confounders, including ethnic background, age of the child, and season of measurement (i.e. basic models). Finally, the independent effects of SEP indicators were assessed by model adjusted for all SEP indicators (i.e. full model). Similar series of multiple logistic regression models were carried out to investigate the associations of ethnic background with both physical activity behaviors. A crude model contained ethnic background only. A basic model was adjusted for confounders, including age of the child and season at measurement. Finally, the full model additionally adjusted for all indicators of family SEP. This stepwise adjustment was used to gain insight into the separate confounding effects of SEP, a construct highly related with ethnic background [25-27]. Child’s sex did not affect the effect estimates; therefore, this variable was left out of the analyses [28]. Collinearity between maternal educational level, paternal educational level, and income were assessed by pair-wise Spearman’s rho correlation coefficients (r > 0.8). Size of the correlation coefficients did not indicate collinearity (0.54-0.60) and therefore these variables were included in the full models simultaneously. To handle missing data in the data, multiple imputation was applied [29]. Five imputed datasets were generated using a fully conditional specified model, thus taking into account the uncertainty of the imputed values. Pooled estimates from these five imputed datasets were used to report effect estimates and their 95% confidence intervals (CIs). Imputations were based on the relationships between all the variables included in this study. All analyses were conducted in 2014 with Statistical Package for Social Sciences (SPSS) version 21.0 for Windows (SPSS Inc., Chicago, IL, USA). A significance level of p < 0.05 was used to indicate significant associations.

### Nonresponse analyses

Children with missing data on both sports participation and outdoor play (n = 1322) were compared with children without missing data (n = 5125) using Chi-square tests. Data were more often missing for children with a low maternal educational level (χ^2^ = 26, df = 3, p < 0.001), children with a low paternal educational level (χ^2^ = 17, df = 3, p < 0.001), children from a low household income (χ^2^ = 23, df = 2, p < 0.001), children with a mother without a paid job (χ^2^ = 4, df = 1, p < 0.05), children with a father without a paid job (χ^2^ = 6, df = 1, p < 0.05), and ethnic minority children (χ^2^ = 470, df = 6, p < 0.001).

## Results

Table 1 shows characteristics of the study population. One third of the children were non-native Dutch (29.4%). Nearly half of children had a mother with a low or mid-low educational level (45.7%). A quarter of children belonged to a household with a net income of less than 2000 euro per month (24.1%). A little over half of the children did not participate in sports (56.1%) and a third of children played outdoors <1 hour per day (33.7%). The prevalence of sports participation was lower, and the prevalence of outdoor play <1 hour per day higher, among low SEP children and ethnic minority children (Table 2).

**Table 1:** **Characteristics of the study population (n = 4726)**

		**Total**	**Missing**
		**n (%)**	**n (%)**
*Family characteristics*			
Maternal educational level	High	124 (26.7)	64 (1.4)
	Mid-high	1285 (27.6)	
	Mid-low	1489 (31.9)	
	Low	643 (13.8)	
Paternal educational level	High	1393 (32.9)	493 (10.4)
	Mid-high	970 (22.9)	
	Mid-low	1138 (26.9)	
	Low	732 (17.3)	
Maternal employment status	Paid job	3342 (75.6)	305 (6.5)
	No paid job	1079 (24.4)	
Paternal employment status	Paid job	3914 (94.2)	569 (12.0)
	No paid job	243 (5.8)	
Household income	> €3200/month	2174 (49.7)	348 (7.4)
	€2000-€3200/month	1148 (26.2)	
	<€ 2000/month	1056 (24.1)	
Ethnic background	Dutch	3338 (70.6)	0
	Surinamese-Creole	163 (3.4)	
	Surinamese-Hindustani	170 (3.6)	
	Dutch Antillean	126 (2.7)	
	Cape Verdean	195 (4.1)	
	Turkish	453 (9.6)	
	Moroccan	281 (6.0)	
*Child characteristics*			
Sex	Girl	2338 (49.5)	0
	Boy	2388 (50.5)	
Age	Months (mean, SD)	73.0 (5.9)	0
Sports participation	Yes	2057 (43.9)	41 (0.9)
	No	2628 (56.1)	
Outdoor play	≥1 hour/day	2587 (66.3)	823 (17.4)
	<1 hour/day	1316 (33.7)	

Table is based on non-imputed dataset.

Values are means (SD) for normally distributed continuous variables and frequencies (percentage) for categorical variables.

**Table 2:** **Meal skipping behaviors according to family socioeconomic position and ethnic background (n = 4726)**

		**Sports participation (n = 4685)**			**Outdoor play (n = 3903)**		
		**Yes**	**No**	p-Value*	**≥1 hour/day**	**<1 hour/day**	p-Value*
		**n (%)**	**n (%)**		**n (%)**	**n (%)**	
Maternal educational level	High	695 (56.0)	545 (44.0)	<0.001	737 (67.4)	356 (32.6)	0.06
	Mid-high	607 (47.6)	667 (52.4)		748 (68.8)	339 (31.2)	
	Mid-low	549 (37.3)	921 (62.7)		791 (64.8)	429 (35.2)	
	Low	184 (28.9)	453 (71.1)		286 (62.7)	170 (37.3)	
Paternal educational level	High	779 (56.4)	602 (43.6)	<0.001	809 (67.5)	390 (32.5)	<0.05
	Mid-high	445 (46.3)	516 (53.7)		586 (70.6)	244 (29.4)	
	Mid-low	409 (36.1)	723 (63.9)		643 (67.5)	310 (32.5)	
	Low	239 (32.9)	487 (67.1)		343 (61.9)	211 (38.1)	
Maternal employment status	Paid job	1548 (46.6)	1773 (53.4)	<0.001	1925 (67.6)	924 (32.4)	<0.01
	No paid job	389 (36.6)	674 (63.4)		514 (62.2)	313 (37.8)	
Paternal employment status	Paid job	1755 (45.2)	2129 (54.8)	<0.001	2229 (67.8)	1061 (32.2)	0.36
	No paid job	79 (32.8)	162 (67.2)		116 (64.4)	64 (35.6)	
Household income	>€3200	1169 (54.2)	988 (45.8)	<0.001	1317 (69.4)	581 (30.6)	<0.001
	€2000- < €3200	410 (36.0)	730 (64.0)		663 (68.3)	308 (31.7)	
	<€2000	329 (31.5)	715 (68.5)		417 (54.2)	352 (45.8)	
Ethnic background	native Dutch	1623 (48.9)	1693 (51.1)	<0.001	2062 (70.9)	846 (29.1)	<0.001
	Surinamese-Creole	64 (39.5)	98 (60.5)		77 (61.1)	48 (38.4)	
	Surinamese-Hindustani	62 (36.9)	106 (63.1)		78 (60.0)	52 (40.0)	
	Dutch Antillean	42 (34.1)	81 (65.9)		59 (63.4)	34 (36.6)	
	Cape Verdean	72 (36.9)	123 (63.1)		74 (51.7)	69 (48.3)	
	Turkish	114 (25.8)	328 (74.2)		143 (45.3)	173 (54.7)	
	Moroccan	80 (28.7)	199 (71.3)		94 (50.0)	94 (50.0)	

Table is based on non-imputed dataset.

*P-Values assessed by Chi-square tests.

Children of mid-high, mid-low, and low educated mothers were more likely not to participate in sports compared with children of high educated mothers, with children of low educated mothers showing the highest odds (OR: 2.73; 95% CI: 2.18,3.42) (basic model, Table 3). Similar results were found for paternal educational level. Children from low income households (OR: 2.18, 95% CI: 1.82,2.61) and middle income households (OR: 1.97; 95% CI: 1.70,2.29) were more likely not to participate in sports compared with children living in high income households. Finally, children of mothers without a paid job were more likely not to participate in sports compared with children of mothers with a paid job (OR: 1.23, 95% CI: 1.06,1.44). Children from low income households had increased odds of outdoor play <1 hour per day compared with children from high income households (OR: 1.32; 95% CI: 1.07,1.64). Independent SEP associations with children’s participation in sports and outdoor play were found for maternal educational level (sports participation and outdoor play), paternal educational level (sports participation), and household income (sports participation and outdoor play) (full model, Table 3).

**Table 3:** **Associations of family SEP indicators with sports participation (no) (n = 4685) and outdoor play (<1 hour/day) (n = 3903)**

	**Sports participation (no)**			**Outdoor play (<1 hour/day)**		
	**Crude model**	Basic model*	Full model**	**Crude model**	Basic model*	Full model**
	**OR (95% CI)**	**OR (95% CI)**	**OR (95% CI)**	**OR (95% CI)**	**OR (95% CI)**	**OR (95% CI)**
*Maternal educational level*						
High (ref)	1.00	1.00	1.00	1.00	1.00	1.00
Mid-high	**1.40 (1.20,1.64)**	**1.33 (1.13,1.55)**	1.07 (0.90,1.27)	0.94 (0.78,1.12)	0.85 (0.70,1.03)	0.85 (0.69,1.04)
Mid-low	**2.14 (1.84,2.50)**	**1.95 (1.65,2.29)**	**1.28 (1.05,1.55)**	1.13 (0.95,1.35)	0.88 (0.72,1.07)	0.82 (0.64,1.04)
Low	**3.17 (2.58,3.89)**	**2.73 (2.18,3.42)**	**1.65 (1.26,2.17)**	1.25 (1.00,1.57)	0.78 (0.60,1.02)	**0.67 (0.49,0.92)**
*Paternal educational level*						
High (ref)	1.00	1.00	1.00	1.00	1.00	1.00
Mid-high	**1.49 (1.26,1.77)**	**1.45 (1.22,1.72)**	**1.25 (1.05,1.50)**	0.88 (0.72,1.08)	0.84 (0.68,1.04)	0.86 (0.68,1.08)
Mid-low	**2.31 (1.97,2.72)**	**2.20 (1.85,2.61)**	**1.59 (1.29,1.95)**	1.04 (0.87,1.24)	0.93 (0.76,1.14)	0.97 (0.76,1.23)
Low	**2.63 (2.19,3.14)**	**2.26 (1.85,2.76)**	**1.46 (1.16,1.83)**	**1.39 (1.13,1.70)**	1.02 (0.80,1.29)	1.05 (0.79,1.40)
*Maternal employment status*						
Paid job (ref)	1.00	1.00	1.00	1.00	1.00	1.00
No paid job	**1.49 (1.30,1.71)**	**1.23 (1.06,1.44)**	1.01 (0.85,1.20)	**1.25 (1.06,1.47)**	0.93 (0.77,1.12)	0.87 (0.70,1.08)
*Paternal employment status*						
Paid job (ref)	1.00	1.00	1.00	1.00	1.00	1.00
No paid job	**1.61 (1.26,2.07)**	1.29 (1.00,1.67)	1.08 (0.82,1.43)	1.29 (0.91,1.82)	0.96 (0.63,1.45)	0.84 (0.54,1.31)
*Household income*						
> €3200/month (ref)	1.00	1.00	1.00	1.00	1.00	1.00
€2000-€3200/month	**2.08 (1.80,2.40)**	**1.97 (1.70,2.29)**	**1.52 (1.28,1.79)**	1.05 (0.88,1.24)	0.97 (0.80,1.16)	1.06 (0.86,1.31)
<€ 2000/month	**2.49 (2.14,2.90)**	**2.18 (1.82,2.61)**	**1.57 (1.27,1.94)**	**1.84 (1.56,2.18)**	**1.32 (1.07,1.64)**	**1.57 (1.21,2.04)**

Table is based on imputed dataset. Bold print indicates statistical significance.

Values represent odds ratios and 95% confidence intervals derived from (multiple) logistic regression analyses.

SEP = socioeconomic position.

*Adjusted for confounders (i.e. ethnic background, child’s age, and season at measurement).

**Additional adjusted for other SEP indicators.

All ethnic minority children were more likely not to participate in sports compared with native Dutch children, with the highest odds for Turkish children (OR: 3.16; 95% CI: 2.51,3.98) (basic model, Table 4). Additional analyses showed that Turkish children did not significantly differ from Moroccan children and Dutch Antillean children (data not shown). Adjustment for family SEP attenuated the associations considerably for all ethnic minority groups, rendering some associations non-significant (i.e. for Surinamese-Creole, Surinamese-Hindustani, and Cape Verdean children) (full model, Table 4). All ethnic minority children were more likely to play outdoors <1 hour per day compared with native Dutch children, with Turkish children displaying the highest odds (OR: 3.56; 95% CI: 2.76,4.58). Turkish children did not significantly differ from Moroccan children and Cape Verdean children (data not shown). Adjustment for SEP attenuated the associations slightly.

**Table 4:** **Associations of ethnic background with sports participation (no) (n = 4685) and outdoor play (<1 hour/day) (n = 3903)**

	**Sports participation (no)**			**Outdoor play (<1 hour/day)**		
	**Crude model**	Basic model* OR	Full model** OR	**Crude model OR**	Basic model* OR	Full model** OR
	**OR (95% CI)**	**(95% CI)**	**(95% CI)**	**(95% CI)**	**(95% CI)**	**(95% CI)**
*Ethnic background*						
Dutch (ref)	1.00	1.00	1.00	1.00	1.00	1.00
Surinamese-Creole	**1.47 (1.06,2.03)**	**1.66 (1.19,2.30)**	1.10 (0.78,1.55)	**1.52 (1.05,2.20)**	**1.61 (1.08,2.39)**	1.48 (0.98,2.24)
Surinamese-Hindustani	**1.64 (1.19,2.26)**	**1.82 (1.31,2.52)**	1.22 (0.87,1.72)	**1.63 (1.13,2.33)**	**1.83 (1.25,2.68)**	**1.73 (1.17,2.57)**
Dutch Antillean	**1.85 (1.27,2.70)**	**2.26 (1.53,3.33)**	**1.51 (1.01,2.26)**	1.41 (0.91,2.16)	**1.87 (1.18,2.95)**	**1.73 (1.08,2.76)**
Cape Verdean	**1.64 (1.22,2.21)**	**1.84 (1.35,2.49)**	1.08 (0.78,1.49)	**2.27 (1.62,3.19)**	**2.45 (1.71,3.50)**	**2.16 (1.47,3.17)**
Turkish	**2.76 (2.21,3.45)**	**3.16 (2.51,3.98)**	**1.92 (1.49,2.47)**	**2.95 (2.33,3.72)**	**3.56 (2.76,4.58)**	**3.55 (2.68,4.69)**
Moroccan	**2.39 (1.82,3.12)**	**2.82 (2.14,3.71)**	**1.70 (1.26,2.30)**	**2.44 (1.81,3.28)**	**2.72 (1.98,3.75)**	**2.59 (1.83,3.66)**

Table is based on imputed dataset. Bold print indicates statistical significance.

Values represent odds ratios and 95% confidence intervals derived from (multiple) logistic regression analyses.

SEP = socioeconomic position.

*Adjusted for basic confounders (i.e. child’s age, and season at measurement).

**Additionally adjusted for all SEP indicators.

## Discussion

In this study, we aimed to assess the associations of family SEP, as indicated by parental educational level, parental employment status, and household income, and ethnic background with children’s sports participation and outdoor play. Low SEP children and ethnic minority children were more likely not to participate in sports and more likely to display low levels of outdoor play compared with high SEP children and native Dutch children, respectively. Associations of family SEP with children’s sports participation and outdoor play differed according to SEP indicator, especially regarding outdoor play.

### Socioeconomic inequalities in sports participation and outdoor play

Our finding of an association between family SEP and children’s sports participation is in line with earlier research that showed low SEP children to participate in organized sports less often compared with high SEP children [9-16]. Congruent with our results, this association was consistently found irrespective of indicator of SEP (e.g. parental educational level, parental occupation, income, or a composite measures of SEP) [9-16]. In contrast, we found that the association between family SEP and children’s outdoor play differed according to SEP indicator, with (only) a low household income predicting low levels of outdoor play. Such inconsistencies between SEP indicators have been observed previously [19], and may account for the discrepant results in previous studies on socioeconomic inequalities in children’s outdoor play [13,18-20]. The results of this study therefore highlight the need for the use of different SEP indicators when investigating socioeconomic inequalities in young children’s physical activity behaviors, outdoor play in particular [30-32].

Reduction of socioeconomic inequalities in children’s sports participation and outdoor play requires knowledge on the underlying pathways [33]. Previous research on the perceived barriers and challenges of engaging in physical activity has shown that low SEP children and their parents experience multiple barriers from different domains, including, but not limited to, time management and scheduling demands, financial barriers, family obligations, lack of adult involvement, lack of control, and environmental barriers (e.g. lack of sports facilities and playgrounds and safety issues) [34-37]. By assessing the independent associations of different SEP indicators with children’s sports participation and outdoor play, the current study may provide some preliminary insights into these different pathways [30-32,38,39].

Independent associations with children’s sports participation were found for parental educational level and household income. A high household income is likely to represent necessary resources for participation in organized sports [32]. In the Netherlands, participation in organized sports involves multiple expenses, including membership fees, costs of sports gear and attributes, and costs associated with transportation [40-42]. Indeed, financial barriers are often mentioned as a major factor restricting sports participation among children from low-income families [35,40-42]. Furthermore, a high income may represent a more favorable residential environment with (quality) sports facilities in the nearby neighborhood [36,37]. With respect to a high parental educational level, we hypothesize that knowledge (e.g. with respect to the health benefits of children’s sports participation), attitudes, and skills (e.g. favorable parenting practices) may represent some of the contributing mechanisms [13,30-32,38]. Low educated parents may also lack the awareness of existent funding opportunities [43], which may help explain why financial barriers remain an important obstacle even in the presence of such funding [35,40-42].

Independent associations with children’s outdoor play were found for maternal educational level and household income. A high income household may indicate the ability to purchase play material (e.g. bicycles and jumping ropes) or may represent a residential environment suitable for children’s outdoor play [32]. Previous studies have shown that favorable attributes of the physical environment, such as access to recreational facilities, presence of sidewalks, controlled intersections, low crime rates, and area affluence positively influence children’s physical activity [44]. With respect to maternal educational level, we hypothesize that low educated mothers may have more free time (e.g. due to unemployment) that enables them to supervise outdoor play of their children [19]. Although maternal employment is often used to capture these work related components of SEP [31,32], the current operationalization (paid job, no paid job) may not have been sufficiently sensitive. We have conducted sensitivity analyses using a more elaborate variable for employment (i.e. no paid job, paid job part-time [<36 hours/week], and paid job full-time [≥36 hours/week]), and found highly similar results (data not shown).

In sum, the finding of independent associations of parental educational level and household income with children’s sports participation and outdoor play indicate different potential pathways relating family SEP to these behaviors. Further research, including both quantitative studies performing formal mediation analyses and qualitative studies, are warranted to provide a deeper understanding of the mechanisms driving the associations of family SEP with children’s participation in sports and outdoor play.

### Ethnic inequalities in sports participation and outdoor play

In accordance with previous research [9,12,17,19], results of the current study showed that ethnic minority children were more likely not to participate in sports and display low levels of outdoor play compared with native Dutch children. Our results disagree with the results of an Australian study that failed to find an association between ethnic background (indigenous versus non-indigenous) and children’s sports participation [15], although this study did find a positive association between main language spoken in home (English) and sports participation. Furthermore, a Danish study found that 6- to 7-year-old non-native Danish children more often played outdoors than Danish children [17]. However, this study did not take into account time spent playing outdoors [17]. In addition to extending the limited evidence base on ethnic inequalities in children’s sports participation and outdoor play, the current study adds by showing that the effects of ethnic background are not uniform across all ethnic minority groups.

Family SEP contributed substantially to the observed ethnic inequalities in sports participation and to a lesser extent to the observed inequalities in outdoor play. These results are in accordance with our finding of more consistent (and more substantial) socioeconomic influences on sports participation than outdoor play. Even though adjustment for family SEP rendered some associations non-significant, we postulate that this may be a consequence of power problems due to low numbers of participants in these groups, rather than full explanation by family SEP.

Over and beyond potential mechanisms related to family SEP, the observed ethnic inequalities may further be explained by variables specifically associated with ethnic minority background, including (amongst others) acculturation characteristics (e.g. language, generational status, length of stay in host country, stressful experiences related to migration and resettlement), religion based norms and values, cultural based norms and values, and discrimination processes [45-47]. For example, parental participation in club-organized sports, as an indicator of experience with and knowledge about the participation in sports, has been shown to be an important mediator in the association between ethnic background and sports participation among children [17]. Also, ethnic minority children more often live in disadvantaged neighborhoods with high crime rates compared with ethnic majority children, which may negatively affect outdoor play due to parental safety concerns or lack of (safe and attractive) physical activity opportunities [48-50].

Previous research in older school-aged children and adolescents has shown that ethnic background may interact with child’s sex in influencing children’s physical activity, sports participation in particular [51,52]. We explored this issue using multiple logistic regression models including interaction terms between ethnic background and child’s sex. Contrary to these previous studies, we did not find significant interaction effects between ethnic background and child’s sex (both p > 0.05, data not shown), suggesting that sex differences in the associations of ethnic background with sports participation and outdoor play are not yet present at such a young age.

### Study strengths and limitations

A major strength of this study is the large sample of children of different socioeconomic and ethnic backgrounds. Several limitations should be considered when interpreting the findings. First, nonresponse analyses showed that low SEP children and ethnic minority children more often had missing data on both physical activity behaviors. Selection bias may have occurred if the associations of family SEP and ethnic background with children’s physical activity behaviors are different for participants and non-participants; however, this is difficult to ascertain.

Second, information bias in the outcome variables may have occurred due to a number of reasons. Because parent-reported questionnaires were used to assess children’s sports participation and outdoor play, social desirable answering (i.e. the over reporting of favorable behaviors) cannot be excluded. Moreover, outdoor play is likely to also occur in settings other than the home environment (e.g. school and after-school care), and therefore parents’ report of outdoor play may have been an underestimation of total outdoor play. Furthermore, data on outdoor play were dichotomized based on the distribution of these data. Existent guidelines for physical activity among youth (5–17 years) specify a recommended amount of at least 1 hour per day of moderate-to-vigorous physical activity [53-56]. As there are no guidelines on outdoor play specifically, we used this cut-off point. However, it should be acknowledged that only parts of outdoor play are spent in moderate-to-vigorous physical activity [57]. Also, the use of a dichotomized variable may have potentially led to a loss of information and statistical power to detect associations. Sensitivity analyses using dichotomized data with a different cut-off point (i.e. 2 hours per day) and continuous data yielded highly similar results (Additional file 1: Table S2).

Third, SEP is a complex, multidimensional construct that can be described and measured in numerous ways [30-32]. In order to assess the association between family SEP and children’s sports participation and outdoor play, we used the commonest individual-level indicators, i.e. educational level, income, and occupation (employment). Inclusion of other individual-level (e.g. housing characteristics) or even area-level indicators (e.g. neighborhood SEP) may have provided further insights into potential mechanisms underlying the observed associations; however, information on these variables was not available for the current study. A related argument concerns the adjustment for family SEP when assessing ethnic inequalities in health and health behaviors. As suggested by other scholars, adjustment for SEP should ideally capture all dimensions of SEP and failure to do so may lead to residual confounding [25-27]. Also, SEP indicators may mediate the effects of other SEP indicators [38]. For example, educational level may influence employment which may in turn influence income. In this case, the latter two indicators would be considered mediators.

Fourth, the results presented in this study were based on a multiple imputed dataset. In addition to preventing loss of information, multiple imputation also deals with potential bias associated with missing data [29]. Sensitivity analyses using complete data showed similar results to the imputed data (Additional files 1: Table S3 and Table S4).

Finally, given that the current results are context specific (e.g. due to the organization of sports and distribution of resources across social groups), caution should be taken when generalizing the current results to other populations.

## Conclusion

Low SEP children and ethnic minority children are more likely not to participate in sports and more likely to display low levels of outdoor play compared with high SEP children and native Dutch children, respectively. These results indicate that low SEP children and ethnic minority children represent important target groups for interventions designed to promote young children’s physical activity. In order to design effective interventions, further research, including qualitative studies, is needed to explore more in detail the pathways relating family SEP and ethnic background to children’s sports participation and outdoor play.

## Additional file

Additional file 1: Table S1. Assessment of sports participation and outdoor play. **Table S2.** Associations of family SEP indicators and ethnic background with outdoor play (n=3903). **Table S3.** Associations of family SEP indicators with sports participation (no) (n=4685) and outdoor play (<1 hour/day) (n=3903). **Table S4.** Associations of ethnic background with sports participation (no) (n=4685) and outdoor play (<1 hour/day) (n=3903).
