# Supplementary material for: Social inequalities in young children’s sports participation and outdoor play
Source: Int J Behav Nutr Phys Act. 2014 Dec 16;11:155. doi: 10.1186/s12966-014-0155-3 (PMC4272790; doi:10.1186/s12966-014-0155-3)
Supplement: Additional file 1: Table S1. — Assessment of sports participation and outdoor play. Table S2. Associations of family SEP indicators and ethnic background with outdoor play (n=3903). Table S3. Associations of family SEP indicators with sports participation (no) (n=4685) and outdoor play (<1 hour/day) (n=3903). Table S4. Associations of ethnic background with sports participation (no) (n=4685) and outdoor play (<1 hour/day) (n=3903). [file 12966_2014_155_MOESM1_ESM.docx]

**Additional file 1**

**Table S1: Assessment of sports participation and outdoor play**

| Questions | Answer categories |
| --- | --- |
| “Does your child take part in sports (for example. football, judo, gymnastics, jazz ballet, tennis, etc)?” | Yes |
|  | No |
| “On average, how many weekdays per week does your child play outside?” | Never on weekdays |
|  | 1 day per week |
|  | 2 days per week |
|  | 3 days per week |
|  | 4 days per week |
|  | Every weekday |
| “On average, how many weekend days per week does your child play outside?” | Never in the weekend |
|  | 1 day in the weekend |
|  | 2 days in the weekend |
| “On the days that your child plays outside, how long, on average, does your child play outside? Differentiate between weekdays and weekend days and answer according to the present season.” | Never |
| (Answer categories for weekdays and weekend days separately) | Less than 30 minutes |
| (Answer categories for mornings, afternoons and evenings after dinner separately) | 30-60 minutes |
|  | 1-2 hours |
|  | 2-3 hours |
|  | 3-4 hours |

**Table S2: Associations of family SEP indicators and ethnic background with outdoor play (n=3903)**

|  | Outdoor play (<2 hours/day) | | | Outdoor play (hours per day) | | |
| --- | --- | --- | --- | --- | --- | --- |
|  | Crude model  OR (95% CI) | Basic model*  OR (95% CI) | Full model**  OR (95% CI) | Crude model  β (95 CI%) | Basic model*  β (95 CI%) | Full model**  β (95 CI%) |
| *Maternal educational level* |  |  |  |  |  |  |
| High (ref) | 1.00 | 1.00 | 1.00 | 0.00 | 0.00 | 0.00 |
| Mid-high | 0.84 (0.69,1.01) | **0.76 (0.63,0.93)** | **0.75 (0.61,0.93)** | **0.11 (0.01,0.21)** | **0.16 (0.07,0.25)** | **0.15 (0.05,0.25)** |
| Mid-low | **0.68 (0.57,0.81)** | **0.55 (0.45,0.68)** | **0.55 (0.43,0.70)** | **0.23 (0.13,0.33)** | **0.35 (0.26,0.45)** | **0.33 (0.22,0.44)** |
| Low | **0.68 (0.54,0.87)** | **0.48 (0.37,0.63)** | **0.49 (0.36,0.68)** | **0.20 (0.07,0.33)** | **0.42 (0.30,0.55)** | **0.40 (0.25,0.55)** |
| *Paternal educational level* |  |  |  |  |  |  |
| High (ref) | 1.00 | 1.00 | 1.00 | 0.00 | 0.00 | 0.00 |
| Mid-high | 0.95 (0.78,1.16) | 0.95 (0.77,1.18) | 1.05 (0.84,1.33) | 0.06 (-0.05,0.17) | **0.25 (0.13,0.37)** | 0.01 (-0.09,0.12) |
| Mid-low | **0.77 (0.65,0.92)** | **0.73 (0.60,0.89)** | 0.91 (0.72,1.15) | **0.19 (0.09,0.30)** | **0.24 (0.14,0.33)** | **0.12 (0.01,0.23)** |
| Low | 0.89 (0.71,1.11) | **0.72 (0.56,0.92)** | 0.96 (0.72,1.28) | 0.09 (-0.03,0.21) | 0.06 (-0.04,0.17) | 0.11 (-0.03,0.24) |
| *Maternal employment status* |  |  |  |  |  |  |
| Paid job (ref) | 1.00 | 1.00 | 1.00 | 0.00 | 0.00 | 0.00 |
| No paid job | 0.89 (0.75,1.05) | **0.72 (0.59,0.87)** | **0.76 (0.62,0.94)** | 0.01 (-0.08,0.10) | **0.13 (0.04,0.23)** | **0.10 (0.01,0.20)** |
| *Paternal employment status* |  |  |  |  |  |  |
| Paid job (ref) | 1.00 | 1.00 | 1.00 | 0.00 | 0.00 | 0.00 |
| No paid job | 1.01 (0.72,1.40) | 0.86 (0.58,1.27) | 0.86 (0.57,1.29) | -0.03 (-0.21,0.16) | 0.14 (-0.04,0.31) | 0.15 (-0.03,0.33) |
| *Household income* |  |  |  |  |  |  |
| > €3200/month (ref) | 1.00 | 1.00 | 1.00 | 0.00 | 0.00 | 0.00 |
| €2000-€3200/month | 0.88 (0.74,1.04) | 0.85 (0.71,1.01) | 1.12 (0.91,1.37) | **0.10 (0.00,0.19)** | **0.12 (0.03,0.21)** | -0.05 (-0.15,0.05) |
| <€ 2000/month | 1.13 (0.95,1.36) | 0.87 (0.70,1.08) | **1.31 (1.01,1.70)** | -**0.14 (-0.24,-0.04)** | 0.05 (-0.06,0.15) | **-0.21 (-0.34,-0.08)** |
| Ethnic background | Crude model  OR (95% CI) | Basic model***  OR (95% CI) | Full model***  OR (95% CI) | Crude model  β (95 CI%) | Basic model***  β (95 CI%) | Full model****  β (95 CI%) |
| Dutch (ref) | 1.00 | 1.00 | 1.00 | 0.00 | 0.00 | 0.00 |
| Surinamese-Creole | 1.21 (0.81,1.79) | 1.30 (0.85,1.97) | 1.49 (0.97,2.30) | -0.19 (-0.40,0.03) | **-0.22 (-0.42,-0.02)** | **-0.32 (-0.52,-0.11)** |
| Surinamese-Hindustani | 1.44 (0.96,2.15) | **1.61 (1.05,2.45)** | **1.86 (1.21,2.87)** | **-0.30 (-0.51,-0.09)** | **-0.35 (-0.54,-0.15)** | **-0.45 (-0.65,-0.25)** |
| Dutch Antillean | 1.13 (0.72,1.78) | 1.54 (0.96,2.46) | **1.85 (1.13,3.02)** | -0.07 (-0.31,0.18) | **-0.24 (-0.47,-0.01)** | **-0.34 (-0.57,-0.10)** |
| Cape Verdean | **1.76 (1.18,2.65)** | **1.91 (1.25,2.92)** | **2.32 (1.48,3.62)** | **-0.40 (-0.60,-0.20)** | **-0.40 (-0.59,-0.22)** | **-0.52 (-0.71,-0.32)** |
| Turkish | **1.63 (1.24,2.14)** | **1.87 (1.40,2.49)** | **2.50 (1.83,3.42)** | **-0.40 (-0.54,-0.26)** | **-0.45 (-0.58,-0.32)** | **-0.60 (-0.74,-0.46)** |
| Moroccan | **2.38 (1.61,2.51)** | **2.78 (1.85,4.17)** | **3.67 (2.38,5.66)** | **-0.51 (-0.68,-0.33)** | **-0.54 (-0.71,-0.38)** | **-0.69 (-0.87,-0.52)** |

Table is based on imputed dataset. Bold print indicates statistical significance.

Values represent odds ratios (95% confidence intervals) derived from (multiple) logistic regression analyses and betas (95% confidence intervals) derived from (multiple) linear regression analyses. SEP=socioeconomic position.

* Adjusted for confounders (i.e. child’s age, and season at measurement, and ethnic background).

** Additionally adjusted for all SEP indicators.

*** Adjusted for confounders (i.e. child’s age and season at measurement).

**** Additionally adjusted for al SEP indicators.

**Table S3: Associations of family SEP indicators with sports participation (no) (n=4685) and outdoor play (<1 hour/day) (n=3903)**

|  | Sports participation (no) | | | Outdoor play (<1 hour/day) | | |
| --- | --- | --- | --- | --- | --- | --- |
|  | Crude model  OR (95% CI) | Basic model*  OR (95% CI) | Full model**  OR (95% CI) | Crude model  OR (95% CI) | Basic model*  OR (95% CI) | Full model**  OR (95% CI) |
| *Maternal educational level* | n=4621 | n=4621 | n=3583 | n=3856 | n=3856 | n=3064 |
| High | 1.00 (ref) | 1.00 (ref) | 1.00 (ref) | 1.00 (ref) | 1.00 (ref) | 1.00 (ref) |
| Mid-high | **1.40 (1.20,1.64)** | **1.32 (1.13,1.55)** | 1.14 (0.95,1.37) | 0.94 (0.78,1.12) | 0.85 (0.70,1.03) | 0.83 (0.67,1.04) |
| Mid-low | **2.14 (1.83,2.50)** | **1.94 (1.65,2.29)** | **1.25 (1.01,1.56)** | 1.12 (0.95,1.33) | 0.88 (0.73,1.08) | 0.83 (0.64,1.07) |
| Low | **3.14 (2.56,3.85)** | **2.71 (2.16,3.39)** | **1.91 (1.37,2.65)** | 1.23 (0.78,1.12) | 0.78 (0.60,1.02) | 0.74 (0.51,1.08) |
| *Paternal educational level* | n=4200 | n=4200 | n=3583 | n=3536 | n=3536 | n=3064 |
| High | 1.00 (ref) | 1.00 (ref) | 1.00 (ref) | 1.00 (ref) | 1.00 (ref) | 1.00 (ref) |
| Mid-high | **1.50 (1.27,1.77)** | **1.46 (1.24,1.73)** | **1.21 (1.00,1.47)** | 0.86 (0.71,1.05) | 0.84 (0.68,1.03) | 0.82 (0.65,1.03) |
| Mid-low | **2.29 (1.95,2.69)** | **2.21 (1.87,2.62)** | **1.52 (1.23,1.87)** | 1.00 (0.83,1.20) | 0.95 (0.77,1.16) | 0.93 (0.72,1.20) |
| Low | **2.64 (2.19,3.18)** | **2.32 (1.89,2.85)** | **1.44 (1.10,1.87)** | **1.28 (1.04,1.57)** | 1.02 (0.80,1.30) | 1.04 (0.76,1.42) |
| *Maternal employment status* | n=4384 | n=4384 | n=3583 | n=3676 | n=3676 | n=3064 |
| Paid job | 1.00 (ref) | 1.00 (ref) | 1.00 (ref) | 1.00 (ref) | 1.00 (ref) | 1.00 (ref) |
| No paid job | **1.51 (1.31,1.74)** | **1.27 (1.09,1.48)** | 0.97 (0.80,1.18) | **1.27 (1.08,1.49)** | 0.94 (0.78,1.13) | 0.94 (0.75,1.18) |
| *Paternal employment status* | n=4125 | n=4125 | n=3583 | n=3470 | n=3470 | n=3064 |
| Paid job | 1.00 (ref) | 1.00 (ref) | 1.00 (ref) | 1.00 (ref) | 1.00 (ref) | 1.00 (ref) |
| No paid job | **1.69 (1.28,2.23)** | **1.36 (1.01,1.82)** | 1.13 (0.80,1.60) | 1.16 (0.85,1.59) | 0.91 (0.64,1.29) | 1.00 (0.68,1.49) |
| *Household income* | n=4341 | n=4341 | n=3583 | n=3638 | n=3638 | n=3064 |
| > €3200/month | 1.00 (ref) | 1.00 (ref) | 1.00 (ref) | 1.00 (ref) | 1.00 (ref) | 1.00 (ref) |
| €2000-€3200/month | **2.11 (1.82,2.44)** | **1.99 (1.71,2.32)** | **1.62 (1.35,1.94)** | 1.05 (0.89,1.24) | 0.96 (0.80,1.16) | 1.01 (0.81,1.26) |
| <€ 2000/month | **2.57 (2.20,3.00)** | **2.28 (1.89,2.74)** | **2.20 (1.69,2.88)** | **1.91 (1.61,2.27)** | **1.36 (1.10,1.69)** | **1.48 (1.09,2.01)** |

Table is based on non-imputed dataset. Bold print indicates statistical significance.

Values represent odds ratios and 95% confidence intervals derived from (multiple) logistic regression analyses.

SEP=socioeconomic position.

* Adjusted for confounders (i.e. ethnic background, child’s age, and season at measurement).

** Additional adjusted for other SEP indicators.

**Table S4: Associations of ethnic background with sports participation (no) (n=4685) and outdoor play (<1 hour/day) (n=3903)**

|  | Sports participation (no) | | | Outdoor play (<1 hour/day) | | |
| --- | --- | --- | --- | --- | --- | --- |
|  | Crude model  OR (95% CI) | Basic model*  OR (95% CI) | Full model**  OR (95% CI) | Crude model  OR (95% CI) | Basic model*  OR (95% CI) | Full model**  OR (95% CI) |
| Ethnic background | n=4685 | n=4685 | n=3583 | n=3903 | n=3903 | n=3064 |
| Dutch | 1.00 (ref) | 1.00 (ref) | 1.00 (ref) | 1.00 (ref) | 1.00 (ref) | 1.00 (ref) |
| Surinamese-Creole | **1.47 (1.06,2.03)** | **1.66 (1.19,2.30)** | 0.95 (0.59,1.53) | **1.52 (1.05,2.20)** | **1.61 (1.08,2.39)** | 1.58 (0.90,2.76) |
| Surinamese-Hindustani | **1.64 (1.19,2.26)** | **1.82 (1.31,2.52)** | 1.36 (0.90,2.05) | **1.63 (1.13,2.33)** | **1.83 (1.25,2.68)** | 1.33 (0.83,2.14) |
| Dutch Antillean | **1.85 (1.27,2.70)** | **2.26 (1.53,3.33)** | 1.23 (0.69,2.21) | 1.41 (0.91,2.16) | **1.87 (1.18,2.95)** | 1.09 (0.54,2.21) |
| Cape Verdean | **1.64 (1.22,2.21)** | **1.84 (1.35,2.49)** | 0.81 (0.52,1.27) | **2.27 (1.62,3.19)** | **2.45 (1.71,3.50)** | **1.88 (1.11,3.18)** |
| Turkish | **2.76 (1.21,3.45)** | **3.16 (2.51,3.98)** | **1.69 (1.25,2.29)** | **2.95 (2.33,3.73)** | **3.56 (2.76,4.58)** | **3.84 (2.75,5.36)** |
| Moroccan | **2.39 (1.82,3.12)** | **2.82 (2.14,3.71)** | 1.33 (0.92,1.92) | **2.44 (1.81,3.28)** | **2.72 (1.98,3.75)** | **2.37 (1.55,3.63)** |

Table is based on non-imputed dataset. Bold print indicates statistical significance.

Values represent odds ratios and 95% confidence intervals derived from (multiple) logistic regression analyses.

SEP=socioeconomic position.

* Adjusted for basic confounders (i.e. child’s age, and season at measurement).

** Additionally adjusted for all SEP indicators.
